# Human olfactory neurosphere-derived cells: a unified tool for neurological disease modelling and neurotherapeutic applications

**DOI:** 10.1097/JS9.0000000000001460

**Published:** 2024-04-23

**Authors:** Saad Irfan, Maudlyn O. Etekochay, Atanas G. Atanasov, Vishnu P. Prasad, Ramesh Kandimalla, Mohammad Mofatteh, Priyanka V, Talha B. Emran

**Affiliations:** a Animal Science Department, Faculty of Animal and Agriculture Sciences, Universitas Diponegoro, Semarang, Indonesia; b PinnacleCare Intl. Baltimore, MD, USA; c Department of Biotechnology and Nutrigenomics, Institute of Genetics and Animal Biotechnology of the Polish Academy of Sciences, Jastrzebiec, Poland; d Ludwig Boltzmann Institute Digital Health and Patient Safety, Medical University of Vienna, Vienna, Austria; e Rajiv Gandhi University of Health Sciences, Jayanagar, Bengaluru, Karnataka; f CSIR-Indian Institute of Chemical Technology Uppal Road, Tarnaka, Hyderabad, Telangana State; g Department of Biochemistry, Kakatiya Medical College, Warangal, Telangana, India; h School of Medicine, Dentistry, and Biomedical Sciences, Queen's University Belfast, Belfast, UK; i Department of Veterinary Microbiology, College of Veterinary Science, Guru Angad Dev Veterinary and Animal Sciences University (GADVASU), Rampura Phul, Bathinda, Punjab, India; j Department of Pharmacy, Faculty of Allied Health Sciences, Daffodil International University, Dhaka, Bangladesh

**Keywords:** neurodegenerative, neuro-infectious, olfactory neurosphere stem cells, Zika virus

## Abstract

As one of the leading causes of global mortality and morbidity, various neurological diseases cause social and economic burdens. Despite significant advances in the treatment of neurological diseases, establishing a proper disease model, especially for degenerative and infectious diseases, remains a major challenging issue. For long, mice were the model of choice but suffered from serious drawbacks of differences in anatomical and functional aspects of the nervous system. Furthermore, the collection of postmortem brain tissues limits their usage in cultured cell lines. Overcoming such limitations has prompted the usage of stem cells derived from the peripheral nervous system, such as the cells of the olfactory mucosa as a preferred choice. These cells can be easily cultured in vitro and retain the receptors of neuronal cells life-long. Such cells have various advantages over embryonic or induced stem cells, including homology, and ease of culture and can be conveniently obtained from diseased individuals through either biopsies or exfoliation. They have continuously helped in understanding the genetic and developmental mechanisms of degenerative diseases like Alzheimer’s and Parkinson’s disease. Moreover, the mode of infection of various viruses that can lead to postviral olfactory dysfunction, such as the Zika virus can be monitored through these cells in vitro and their therapeutic development can be fastened.

## Introduction

Highlights

- Alzheimer’s ranked third, and motor neuron diseases.
- This review emphasizes on the application of olfactory neurosphere-derived cells.
- Olfactory stem cells experience a change in function from neuro-regeneration.
- Human olfactory neurospheres derived cells are valuable models for studying Parkinson’s disease.

Degenerating neurons of the brain and nervous system are one of the prominent reasons for the social and economic burden on patients and the healthcare system around the world^1^. In 2016, neurological diseases were a major cause of disability globally^1^. Out of these, neurodegenerative diseases, such as Alzheimer’s ranked third, and motor neuron diseases, such as Parkinson’s ranked 11th^1^. Despite ongoing clinical trials aiming to develop effective treatments for such neurodegenerative diseases over the past decades, curing such diseases is not completely possible^2^.

In addition, the nervous system is prone to various viral infections, with severe consequences^3,4^. Olfactory nerves represent one of the shortest routes for foreign particles making them susceptible to several viruses, such as West Nile virus, Zika virus, and SARS-CoV-2^3–5^, leading to postviral olfactory dysfunction. Studying them from the deep-rooted level and development of therapeutics is of utmost necessity to protect children and adults from life-long impairments^5^.

A major challenge is developing effective disease models to understand the complexity of these diseases of the neuronal system^6^. The foremost characteristic feature of a disease model is a system that can display a full set of the pathological features underlying a disease^6^. Recently, the use of mouse models has revealed various setbacks in studying neurodegenerative diseases due to significant differences in anatomical and physiological parameters, neurotransmitter action, metabolic features, and therapeutic responses between Humans and Mice^7^. Thus, the use of human cell lines in neurological disease modeling has become inevitable^6,7^.

A major problem in applying human cell lines in neurological research is a lack of a large quantity of neuronal cell material from living individuals^8^. Generally, these cells are obtained from progenitors of neuronal cells and neurons generated from pluripotent cells^6^. However, this poses a huge problem of small sample size with hereditary and highly polygenic complications^8^. In addition, stem cells of neuronal origin from the matured brain are only available for research purposes postmortem^8^. These hindrances mark stem cells of the olfactory neuroepithelium as a possible alternate, which can restore the olfactory receptors life-long^9^. Moreover, they can be obtained through nasal biopsies or even through noninvasive procedures and can be cultivated in vitro^9^. More interestingly, direct cultivation from neurospheres grown from nasal biopsies is possible^9,10^. Cells of the olfactory neuroepithelium are gaining huge demand as model systems for neurodegenerative and neuroinfectious disease research and the development of therapeutics^10^.

This review emphasizes on the application of olfactory neurosphere-derived cells from humans as a model to study neurological diseases of degenerative and infectious nature and for neurotherapeutic applications necessary for drug discovery. This literature review was performed by searching literature on the hallows of modeling and therapeutics of neurodegenerative and neuroinfectious disorders. The role of olfactory neurosphere-derived cells as disease models was searched through various sources such as research articles, systematic and literature review articles, and books. Search keywords including neurodegenerative, Alzheimer’s disease, dementia; neuro-infectious, infectious brain diseases and olfactory neurospheres, olfactory stem cells, neurospheres, neural stem cells were used. The limitations of the review are the need for more scientific research data on the specific use of olfactory neurosphere-derived cells for disease modeling and therapeutics. Moreover, data on clinical trials for implications are limited. Notable advantages of olfactory neurospheres compared to traditional models have been discussed alongside the various solutions to overcome drawbacks.

### Human olfactory 3D neurosphere-derived cells

**Figure 1:**
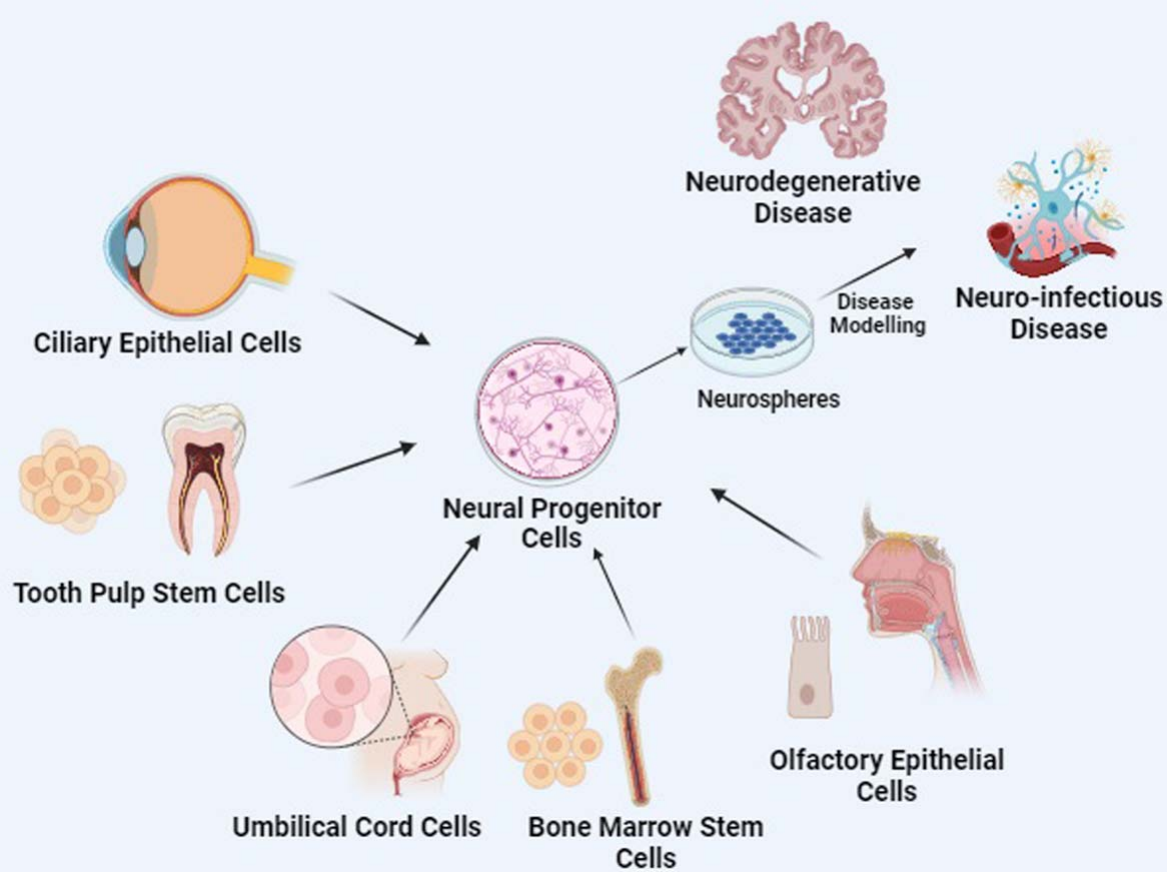
Various neural progenitor cells that can be utilized for creating 3D neurospheres used for disease modeling. Figure created with BioRender.com.

Human from three-dimensional (3D) neurospheres or spheroids are cell lineages that can be prepared from cell structures consisting of progenitor cells or terminally differentiated neurons^11^. The ciliary epithelium, tooth pulp, human blood from the umbilical cord, mesenchymal stem cells of the bone marrow, and more recently, olfactory epithelial cells, constitute a few of the neuronal precursors from which these human neurospheres can be derived^12^ (Fig. 1). 3D neurospheres represent an advanced structural and functional design compared to traditional 2D cultures, featuring dynamic interactions among cells and their extracellular matrix^13^.

### 3D Neurospheres from olfactory mucosa: features, culture, and development

**Figure 2:**
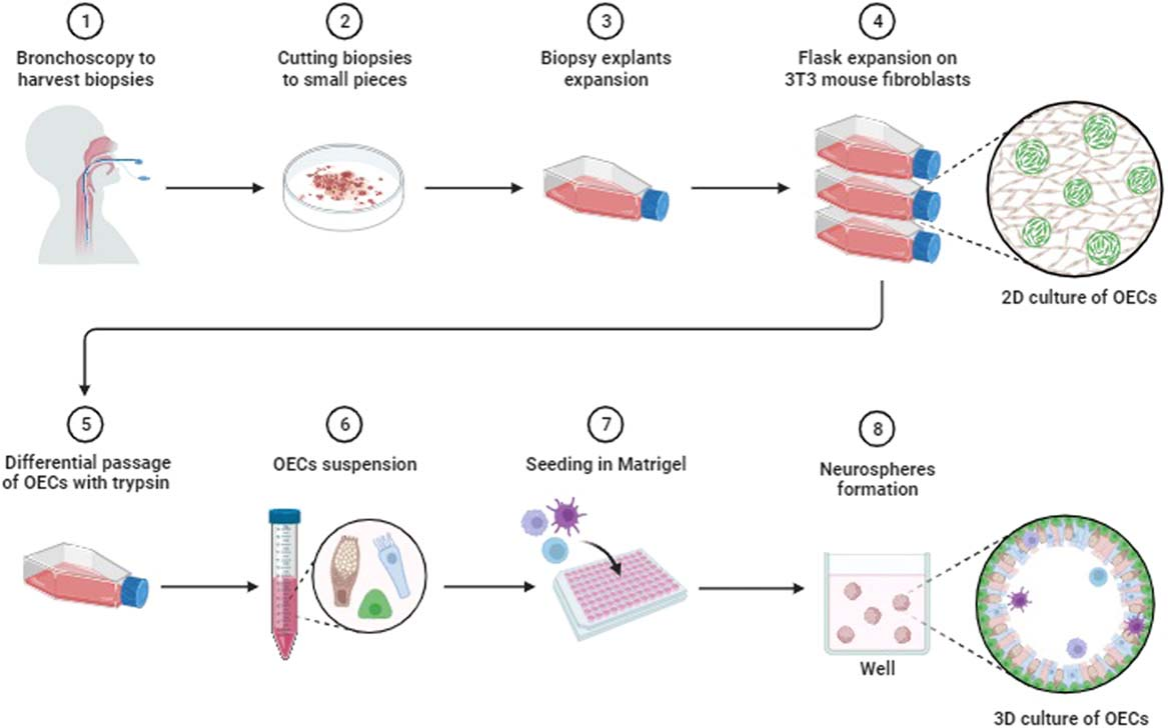
Culture of human olfactory epithelium-derived 3D neurospheres from nasal biopsies. Figure created with BioRender.com.

The advent of pluripotent stem cells derived from humans has significantly enhanced our ability to model neurodegenerative diseases, providing an unlimited source of cells for neurotherapy^6^. The neural tissue of olfactory mucosa is easily acquired from human adults and can demonstrate disease-specific changes representing many neurodegenerative and neuroinfectious diseases^14^. These cells can be obtained from patient biopsies that can be grown into neurospheres within the serum-containing medium as confluent cells capable of long-term storage in very low-temperature conditions (Fig. 2)^15^.

Olfactory mucosa is comprised of multipotent cells that can be propagated as neuronal progenitor cells in neurospheres capable of differentiating between neuronal and glial cells^16^. These cells exhibit changes in viscoelastic behavior with plastic deformation and mechanical properties dependent on their sizes, whereas smaller ones are stiffer, indicating their mechanical strengths under oxidative stress^17^.

Studies on developmental potential and plasticity have revealed that they can integrate, proliferate, and move with analogous kinetics similar to embryonic stem cells, to the olfactory bulb, to develop similar electrophysiological characteristics while reacting to diverse neurogenic stimuli to become functional neurons in the brain^18^.

Studies by Roisen *et al*.^19^ and Winstead *et al*.^20^ report that around 75 different neurosphere-forming cell lines were successfully established from primary cultures obtained from patients. In a separate study, Jiménez-Vaca and colleagues^21^ investigated the differentiation of neurospheres derived from human olfactory epithelial cells to assess their multipotency. These findings on the potential of differentiation indicated that differentiation the process could be easily induced in response to changes in the growth media, particularly in media devoid of epidermal and fibroblast growth factors^19–21^.

### Noninvasive isolation and surface characterization of olfactory neurospheres

In addition to invasive methods, such as biopsies, human olfactory neurospheres can be obtained through nasal exfoliation^21^. Exfoliated cells derived from the septal and lateral cavities of the olfactory region can be cultured in antibiotics-containing media. Such cell exfoliates are dissociated mechanically after collection and the pellet acquired through centrifugation is re-suspended in a serum-free medium and kept for propagation to primary neurospheres within 4–7 days^22^.

**Figure 3:**
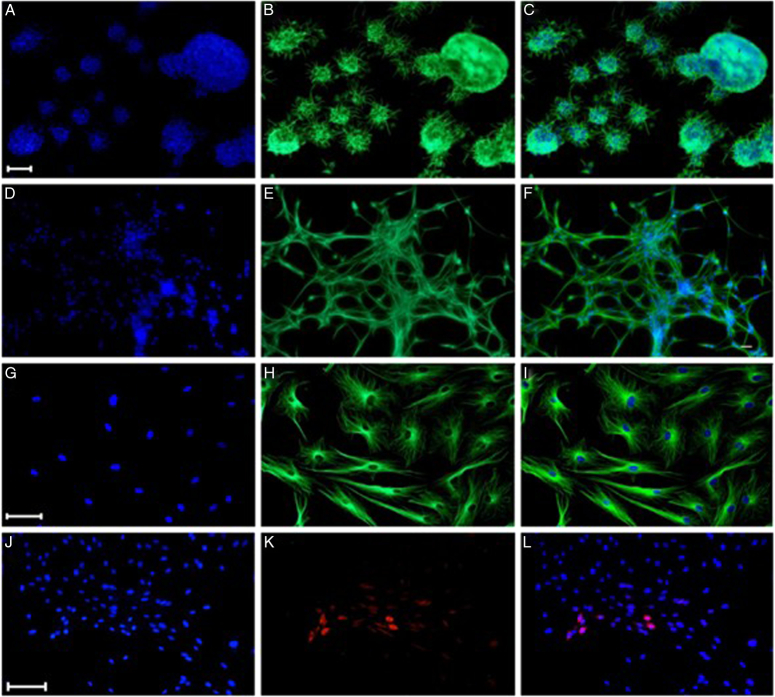
Differentiation of Neurospheres grown in vitro independent of growth factors that initiate differentiation. DAPI-epithelial cells (A), differentiated neurospheres epifluorescence (D, G, J), βIII-tubulin immune-stained neurospheres (B), 7-day-old differentiated neurospheres within factors (E), Double-labeled (βIII-tubulin and DAPI) neurospheres (C), differentiated neurospheres (F), Stem cell cultures in passage 28 (G, J), βIII-tubulin antibody (H), anti-GFAP antibody (K), merge (I, L), and DAPI stained (G, J). Figure reproduced with permission from: https://link.springer.com/article/10.1007/s12035-017-0500-z.

Immunocytochemistry methods using various neural progenitor markers revealed that neurospheres expressed markers, such as nestin, SOX2, and β-III tubulin with similar signal intensity (Fig. 3). The neurospheres composed of progenitor cells were mostly undifferentiated, capable of being propagated, and maintained through several passages^21^. Neurospheres cultured in a medium containing serum but devoid of growth factors were differentiated into neurons, with some differentiating into glial cells^21^.

### Modeling neurological diseases: advantages and drawbacks of olfactory neurosphere

**Figure 4:**
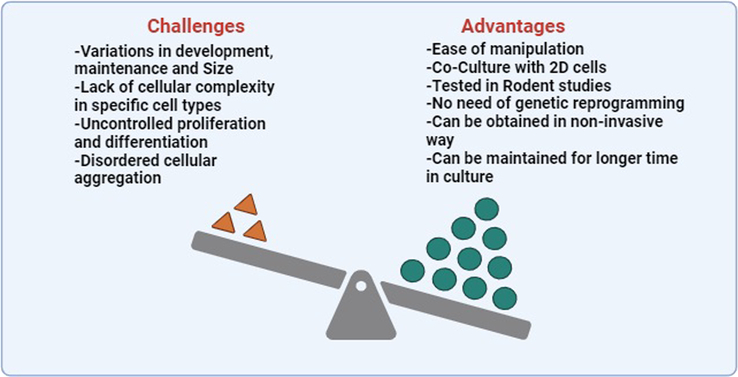
Advantages and challenges in the path of application of human olfactory neurospheres in disease modeling. Figure created with BioRender.com.

A major challenge in the modeling of neurological diseases is the smaller sample size and the requirement to acquire postmortem cells from^8^. Moreover, incorporating animal models in such studies suffers from serious drawbacks like nonhomology and differences in complexity and neurotransmitter function^7^. All these setbacks have prompted the development of disease models for neurodegenerative diseases from olfactory neurosphere-derived stem cells that have several advantages as models^21^. Figure 4 summarizes the advantages and challenges associated application of human olfactory neurospheres in disease modeling.

### Advantages of olfactory neurosphere-derived stem cells

One notable advantage associated with using in vitro neurospheres as models for the human central nervous system is the control over internal and external stimuli^12^. Notably, neurospheres derived from the olfactory system can be cultured along with 2D cells and other acellular matrices, thereby offering contact-mediated stimuli for growth and differentiation^18^. Such combined cultures guide neurospheres towards specific phenotypes, as well as enhance their regenerative properties that are vital for therapeutic applications^23^. Such an interaction was demonstrated in an adipose stem cell-derived neurosphere co-cultured with acellular dermal matrix as an effective therapeutic option for repairing injuries of the peripheral nerves, compared to neurospheres in isolation^24^.

Moreover, some studies have investigated the use of neurospheres in rodent transplantation, aiming to achieve target tissue regeneration in degenerative diseases, such as Alzheimer’s, macular degeneration, and spinal cord injuries. Notably, neurospheres have a higher potential to restore original cell lineages after transplantation in comparison to transplantation of undifferentiated cells, resulting in behavioral and cognitive improvement in diseased model organisms^25,26^. Indeed, neurosphere cultures represent a platform to examine fetal and adult central nervous system in vitro in combination with maintaining their integrity to study developmental processes and therapeutic intervention^12^.

Moreover, neurospheres derived from this region are capable of long-term self-maintenance in growth media^27–29^. The utilization of such cells facilitates the collection of a substantial number of individual samples, which proves invaluable for enhancing our understanding of disease etiology, diagnostic procedures, and drug discovery efforts^29^. Apart from all these advantages, certain limitations need to be considered with the application of olfactory neurospheres for disease modeling and neurotherapeutics.

### Challenges in the implication of olfactory neurosphere-derived stem cells and the way out

In contrast to its advantages, olfactory neurosphere technology comes with several limitations that hinder its prominence in disease modeling. These drawbacks encompass disparities in development and maintenance, a relative lack of cellular complexity, unregulated proliferation and differentiation, irregular cellular aggregation, and the absence of precise and standardized high-throughput compatible assays for drug screenings^30^. Furthermore, when juxtaposed significant differences exist between human and rodent studies about the appearance of cells, expression of lineage markers, and responses to growth factors^31^.

One such key problem that appears during culture is the lack of equilibrium between neural and non-neural cells, as sometimes neurosphere-derived cells are generated as a mixture of stem cells^32^. However, recent developments in the field have enabled channeling cell development towards desired sub-types through protocol modification^33^. Moreover, undifferentiated stem cells from neurospheres are not stable, and minute variations in their handling protocol may lead to totally different results^33^. Overcoming these challenges through standardization and maintenance of proper culture environment and monitoring strategies can represent olfactory neurosphere-derived stem cells as unified tools for disease modeling and neurotherapeutics in neurodegenerative and neuroinfectious diseases.

### Olfactory neurosphere-derived cells used in neurodegenerative diseases

**Figure 5:**
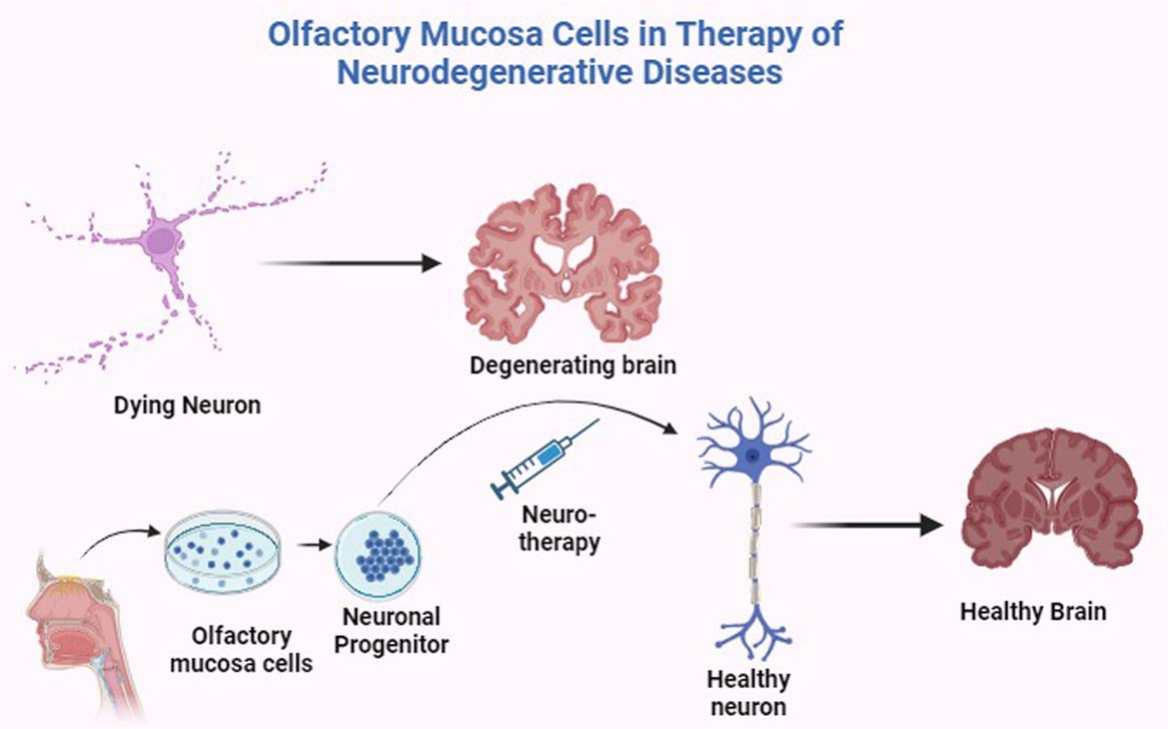
Use of Olfactory mucosa derived stem cells from humans for treatment of neurodegenerative diseases. Figure created with BioRender.com.

The olfactory mucosa stands out as a more pertinent choice when considering neural tissue compared to other progenitor cells, primarily due to the distinctions in cell functions and tissue structure^14^. These cells introduce novel models to study diseases linked to the nervous system, and they hold applicability to all adults, thereby opening new avenues for comprehending the pathology of intricate disorders^18^. Notably, these cells can be cultured using standard protocols, preserved through freezing and banking, thawed on-demand, and expanded in quantity for comprehensive analyses of gene and protein expression^21^. They have shown great prospects in the modeling of neurodegenerative diseases where neuronal connections are lost with disease progression^34^. Furthermore, these cells can be harnessed for therapeutic purposes in treating diseases where the production of neuronal progenitor cells is essential such as seen in the case of spinal cord injury^35^ (Fig. 5).

### Alzheimer’s disease

Currently, more than 55 million people globally are suffering from dementia, with Alzheimer’s disease being the most prominent, accounting for ~60–70% of the cases^36^. Alzheimer’s is a progressive disorder that primarily affects the entorhinal cortex and hippocampus, structures associated with memory, and cognitive abilities^34,37^.

3D olfactory neurosphere-derived cells can serve as a valuable model for Alzheimer’s disease, representing disease-related variations^34^. Studies employing this model have contributed to the identification of potential genes, such as A-Kinase Anchoring Protein 6 (AKAP6), which warrant further investigation^34^. RNA sequencing of neurosphere-derived cells of olfactory origin from Alzheimer’s disease patients has provided valuable insights into the expression patterns of markers associated with neuronal-glial cell differentiation, offering a novel model to investigate alterations in early Alzheimer’s disease-related pathways^38^. Extensive gene pathway analysis has uncovered disruptions in various cellular processes with ageing, and cognitive decline in neurosphere cells of nasal mucosa of affected individuals^34^. Importantly, such studies have revealed that neurosphere-derived cells obtained from olfactory mucosa are characterized by alterations related to cognitive functions and other aspects of brain function^35–37^.

Olfactory neurospheres can replicate oxidative stress associated with Alzheimer’s disease^37^. Notably, the use of fluorescence lifetime imaging microscopy has significantly aided in detecting oxidative alterations with a minor impact on cellular physiology^37^. This innovative approach has the caliber to expedite the formulation of antioxidant-based therapeutics, that can contribute to detecting early symptoms of this disease.

Furthermore, several factors, have been found in the neurospheres from Alzheimer’s disease to influence the development of stem cells towards neurones. Compounds, such as retinoic acid, forskolin, and sonic hedgehog function as neurological growth promoters^39^. These results further support the potential application of olfactory neuroepithelial progenitors in the treatment of degenerative diseases of the neurones like Alzheimer’s.

### Parkinson’s disease

Parkinson’s disease is the second-leading neurodegenerative disease globally, following Alzheimer’s^40^. It is characterized by tremors, rigidity, and mobility challenges, while cognitive decline often becomes evident in later stages^40^. Conditions like sporadic Parkinson’s disease pose modeling challenges due to intricate interactions of unknown environmental stimuli and unidentified risk factors of gene level. To circumvent these challenges, readily available cells such as lymphocytes and skin fibroblasts have been employed to determine distinctions between patients and controls. Regrettably, these cell types are not adequately able to reflect the tissue-specific dissimilarities essential for the working of the brain^41^.

Human olfactory neurospheres provide a means to investigate the impact of environmental stimuli on genetically diverse cells concerning these neurodegenerative diseases to shed light on the interaction between genes and the environment^6^. These cells promise the treatment of Parkinson’s disease is attributed to their ability to thrive, produce dopamine, and offer neurotrophic support in toxin-affected environments, including the neurotoxic conditions found in the Parkinsonian brain^42^. Furthermore, these cells present a considerable advantage as an autologous cell source, eliminating the wait for histocompatible donors and sparing patients from the application of immunosuppressive drugs required at post engraftment stage^42^.

Most cases of Parkinson’s disease are idiopathic and arise from a confluence of various gene and environment-related factors^43^. Immunophenotypic heterogeneity in olfactory neurosphere-derived cell cultures from diseased individuals has been revealed by flow cytometry studies^43^. While antibodies to nestin and OCT4 labeled a smaller number of cells at lower intensity compared to antibodies to CD105 and CD73, the majority of cells displayed immunopositivity for both of these markers^43^. According to a study by Matigian *et al*., the cells showed positive signals for neural progenitors and growing neurons, such as TUB3 (β-tubulin III), but negative signals for SOX2 and other proteins linked to more differentiated phenotypes, such as CD45 and GFAP. Cell lines derived from human olfactory neurospheres have anomalies in biological processes, involving altered mitochondrial function, oxidative stress production, and pollutant metabolism^15^. According to research by Cook and colleagues^44^, NRF2 activation can address metabolic deficits attributed to the condition, which could widen the door for NRF2-based Neurotherapy for Parkinson’s disease. In summary, cells derived from human olfactory neurospheres are valuable models for studying Parkinson’s disease.

### Olfactory neurosphere models in neuroinfectious diseases

Neuroinfectious diseases are conditions of the nervous system induced by pathogens^5^. The olfactory system is susceptible to various viruses, including West Nile virus, Japanese encephalitis virus, Zika virus, and more recently, SARS-CoV-2, which can lead to olfactory dysfunction^4,5^. However, there is a significant shortage of suitable models for studying these diseases.

One such virus is the congenital Zika syndrome (CZS)-causing Zika virus (ZIKV), a flavivirus. A broad spectrum of clinical symptoms has been identified in CZS, such as speech delays, movement or coordination problems, hearing and vision loss, intellectual disability, and speech impairment^45^. It had been long unknown if ZIKV can affect the olfactory system and result in PVOD. In addition, little was understood about the characteristics, range, and major targets of ZIKV inside the olfactory system^5^.

**Figure 6:**
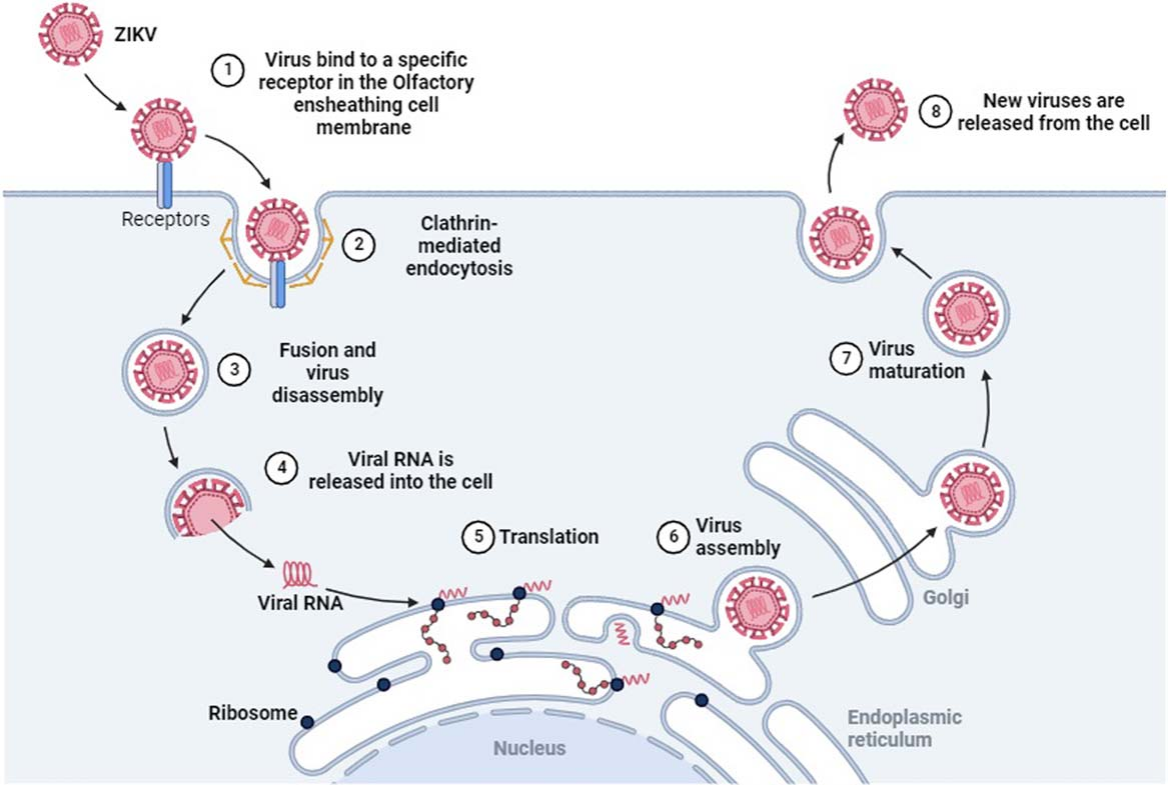
Mechanism of infection of Zika virus into Olfactory mucosa cells as understood through studies on Olfactory 3D neurospheres. Figure created with BioRender.com.

Studies using human olfactory neurospheres made from patient olfactory mucosa have helped in understanding the sensitivity and biological reactions generated by the olfactory system to this viral infection^5^. The results show that ZIKV effectively multiplies in olfactory tissues and primarily targets nasal ensheathing cells^5^. ZIKV shows an extensive cellular tropism, co-localizing in some populations of basal cells, mature or immature olfactory neuronal populations, and sustentacular cells that reside within the olfactory epithelium^45^. Significant antiviral immune responses are produced by ZIKV infection in the nasal mucosa and bulb tissues, leading to the amplification of antiviral response-related genes and proinflammatory cytokines/chemokines^5^. Histopathological and transcriptome analyses for the olfactory receptor system have demonstrated typical tissue damage^45^. The findings suggest that olfactory system disorders are triggered by ZIKV infection (Fig. 6). Moreover, ZIKV promotes the release of various cytokines in the olfactory bulb and mucosa, which, in turn, results in an imbalance of multiple host genes and ultimately impacts the development of an infection within the olfactory system^5^.

In certain investigations, the process underlying the olfactory loss associated with chronic rhinosinusitis has been elucidated via the utilization of olfactory mucosal cells collected from patients^46^. According to studies by Chen *et al*.^46^, olfactory stem cells experience a change in function from neuro-regeneration to immune-mediated defense due to persistent inflammation. Even though the cells of nasal mucosa include longstanding, strongly regenerative basal stem cells in chronic inflammatory rhinosinusitis severely compromise the human olfactory neuroepithelium. Chronic inflammation initiates basal cell-mediated regeneration and damages olfactory neurons while maintaining basal cells within an undifferentiated state. Research on loss-of-function of cells has demonstrated that basal cells promote inflammatory signaling in a manner reliant on NF-kB, which in turn boosts T cell and macrophage local proliferation. The mechanism behind the olfactory loss is attributed to chronic rhinosinusitis, which is brought on by a shift to immune defense from regeneration inside epithelial stem cells of the mucosa^46^.

### Future prospects and conclusion

Human 3D olfactory neurosphere models, both in vitro and ex vivo, show great promise for satisfying the growing demands of neuroscience for more sophisticated modeling platforms^5^. Due to their adaptability and homology, these neurospheres are ideal instruments for studying neurodegenerative and neuroinfectious diseases, as well as helping in the development of translational therapies for these conditions^6^. However, despite the enormous potential of human 3D neurospheres, several issues about their maturity, reproducibility, and variability should be solved before their application in translational biomedical research^47,48^. Ideally, automated liquid and plate managing systems would mitigate concerns about culture variability by standardizing changes to the medium, the formation of the embryoid body, and the distribution of vital nutrients in the cultures^15^.

When specific and standardized biocompatible substances are paired with 3D printing technology, the reproducibility of these models for the study of diseases offers promise^49,50^. Elegant methods for extended cell culture and technologies for accelerating the division of cells and growth demonstrate promise in maturation induction^51^. Olfactory neurosphere-derived cells have been regarded as next-generation models for studying neurodegenerative disorders because they have many advantages over embryonic or induced pluripotent stem cells^6^. They also offer a flexible platform for studying different infectious diseases of the nervous system, the underlying causes of these diseases, and possible treatment options^45^.

The prospects are further important to be understood from the eyes of artificial intelligence. Studies have shown how ChatGPT can aid in providing real-time information on the outbreak of infectious diseases around the globe, including neuroinfectious diseases^52^. Scientists can be prompted to investigate the mechanism of infection and develop vaccines if disease models are readily available^52^. Furthermore, with the aid of ChatGPT, clinical cases involving neuro-anatomical abnormalities or pathologies can be presented. Practitioners can analyze the cases and apply their knowledge with neurospheres to propose treatment options^53^. Such collaboration with artificial intelligence holds great prospects in bringing in neurospheres to the study and therapeutics of neurological disorders^52,53^. The future approaches need to focus on in-vivo clinical trials to bring this as the savior to neurodegenerative and neuroinfectious diseases leading to therapeutic interventions and drug discovery.

## Ethical approval

Not applicable.

## Sources of funding

No funding was received.

## Author contribution

S.I. and M.O.E.: writing original draft; A.G.A. and V.P.P.: data collection and editing; R.K.: editing and revision; M.M. and P.V.: critical review and revision; T.B.E.: data collection, revision, and supervision.

## Conflicts of interest disclosure

Authors declare that they have no conflicts of interest.

## Research registration unique identifying number (UIN)

1. Name of the registry: not applicable.
2. Unique identifying number or registration ID: not applicable.
3. Hyperlink to your specific registration (must be publicly accessible and will be checked): not applicable.

## Guarantor

Talha Bin Emran.

## Provenance and peer review

Not commissioned, internally peer-reviewed.
